# The Evolution and Disparities of Online Attitudes Toward COVID-19 Vaccines: Year-long Longitudinal and Cross-sectional Study

**DOI:** 10.2196/32394

**Published:** 2022-01-21

**Authors:** Chunyan Zhang, Songhua Xu, Zongfang Li, Ge Liu, Duwei Dai, Caixia Dong

**Affiliations:** 1 Institute of Medical Artificial Intelligence The Second Affiliated Hospital of Xi'an Jiaotong University Xi'an China; 2 School of Mathematics and Statistics Xi'an Jiaotong University Xi'an China

**Keywords:** COVID-19, vaccine, attitude, Twitter, data mining, pandemic, population group, evolution, disparity

## Abstract

**Background:**

Due to the urgency caused by the COVID-19 pandemic worldwide, vaccine manufacturers have to shorten and parallel the development steps to accelerate COVID-19 vaccine production. Although all usual safety and efficacy monitoring mechanisms remain in place, varied attitudes toward the new vaccines have arisen among different population groups.

**Objective:**

This study aimed to discern the evolution and disparities of attitudes toward COVID-19 vaccines among various population groups through the study of large-scale tweets spanning over a whole year.

**Methods:**

We collected over 1.4 billion tweets from June 2020 to July 2021, which cover some critical phases concerning the development and inoculation of COVID-19 vaccines worldwide. We first developed a data mining model that incorporates a series of deep learning algorithms for inferring a range of individual characteristics, both in reality and in cyberspace, as well as sentiments and emotions expressed in tweets. We further conducted an observational study, including an overall analysis, a longitudinal study, and a cross-sectional study, to collectively explore the attitudes of major population groups.

**Results:**

Our study derived 3 main findings. First, the whole population’s attentiveness toward vaccines was strongly correlated (Pearson *r*=0.9512) with official COVID-19 statistics, including confirmed cases and deaths. Such attentiveness was also noticeably influenced by major vaccine-related events. Second, after the beginning of large-scale vaccine inoculation, the sentiments of all population groups stabilized, followed by a considerably pessimistic trend after June 2021. Third, attitude disparities toward vaccines existed among population groups defined by 8 different demographic characteristics. By crossing the 2 dimensions of attitude, we found that among population groups carrying low sentiments, some had high attentiveness ratios, such as males and individuals aged ≥40 years, while some had low attentiveness ratios, such as individuals aged ≤18 years, those with occupations of the 3rd category, those with account age <5 years, and those with follower number <500. These findings can be used as a guide in deciding who should be given more attention and what kinds of help to give to alleviate the concerns about vaccines.

**Conclusions:**

This study tracked the year-long evolution of attitudes toward COVID-19 vaccines among various population groups defined by 8 demographic characteristics, through which significant disparities in attitudes along multiple dimensions were revealed. According to these findings, it is suggested that governments and public health organizations should provide targeted interventions to address different concerns, especially among males, older people, and other individuals with low levels of education, low awareness of news, low income, and light use of social media. Moreover, public health authorities may consider cooperating with Twitter users having high levels of social influence to promote the acceptance of COVID-19 vaccines among all population groups.

## Introduction

### Background

Since the emergence of the COVID-19 pandemic in 2019, human health and life have been gravely jeopardized globally. Governments and public health agencies worldwide primarily implemented the following 2 measures to control this pandemic: (1) nonpharmaceutical preventive methods, such as social distancing [1], and (2) COVID-19 vaccine development and mass vaccination to achieve herd immunity [2]. However, implementing nonpharmaceutical interventions is only a short-term solution since it will seriously affect the development of society. Vaccines, on the other hand, are more effective against infectious diseases.

Traditionally, developing a new vaccine from scratch is a complex process, which takes considerable time to accomplish. The main procedures of traditional vaccine development include preclinical studies (about 2-4 years); phase I, II, and III trials (about 5-7 years total); and manufacturing and approval (about 1-2 years) [3]. However, due to the great urgency of the COVID-19 pandemic, vaccine production was accelerated by shortening and paralleling the vaccine development steps. Although all usual safety and efficacy monitoring mechanisms were guaranteed to remain in place, varied attitudes toward these new vaccines have arisen among different population groups. Therefore, it is essential for us to study the evolution and disparities of attitudes across population groups accompanying the introduction of COVID-19 vaccines.

### Literature Review

**Figure 1:**
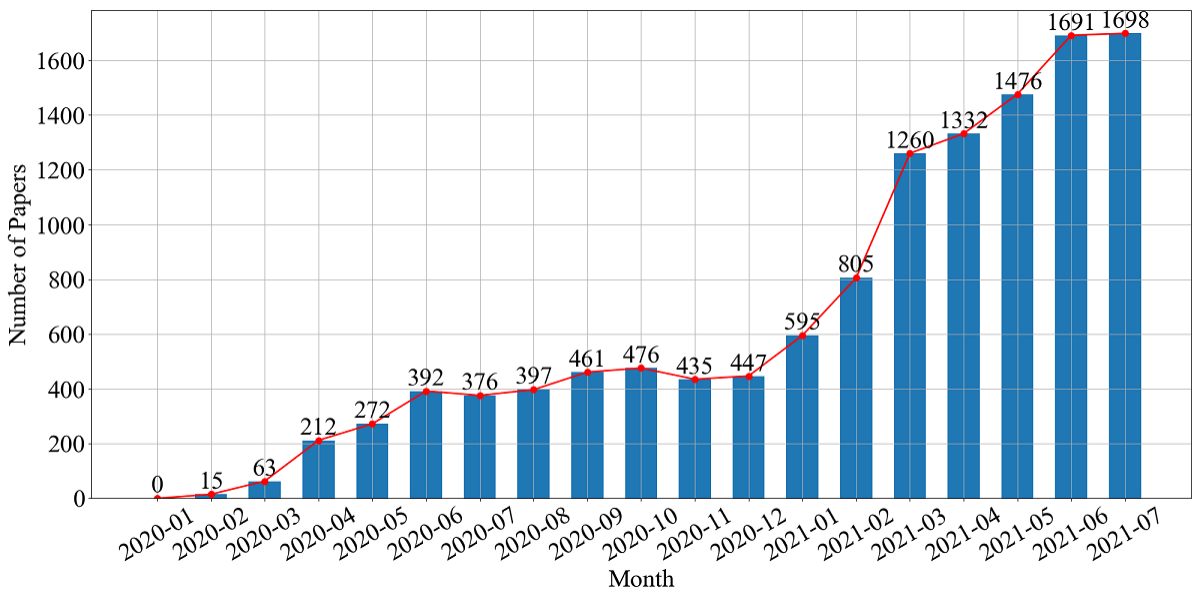
Monthly statistics on newly published papers related to COVID-19 vaccines.

To identify COVID-19 vaccine-related literature, we searched the World Health Organization COVID-19 database [4] with the keywords “vaccine” and “vaccination.” This database is a comprehensive multilingual source of COVID-19 literature from various bibliographic databases (including MEDLINE, PubMed, and Scopus), hand searching, and the addition of other expert-referred scientific articles. Through filtering out preprints, choosing papers written in English, and excluding papers with incomplete information, a total of 12,403 papers were retrieved from the outbreak of the pandemic to July 31, 2021. Then, we counted and plotted the number of papers published each month, as shown in Figure 1. Since the first COVID-19 vaccine-related paper was published in February 2020, the number of papers grew fast until June 2020, and then remained steady from June to December 2020. After January 2021, it increased sharply again.

In order to better understand the current research state of public attitudes toward COVID-19 vaccines, we filtered papers with the term “attitude” in the titles, and “cross-sectional” or “longitudinal” in the titles or abstracts, and retrieved 85 relevant papers. Then, we identified the data collecting methods used in these studies manually, and discovered that there were primarily 2 types as follows: survey (81 papers, 95%) and data mining (4 papers, 5%). Studies involving surveys mainly adopted a cross-sectional design to investigate public attitudes toward COVID-19 vaccination over a short period, while studies involving data mining employed either a longitudinal or cross-sectional design, but rarely both. We conducted a detailed literature review of COVID-19 vaccine-related studies involving these 2 frequently used analysis methods (cross-sectional and longitudinal).

A cross-sectional study analyzes data collected from a population or a predefined subset at a single point in time. Many studies have used this method, primarily through surveys, to explore populations’ attitudes toward receiving COVID-19 vaccination and the factors that affect these attitudes. For example, Lazarus et al [5] surveyed individuals randomly across 19 countries in June 2020. They concluded that the levels of willingness to accept a COVID-19 vaccine were insufficient to meet the requirements for community immunity in most of the 19 countries. Many cross-sectional studies discerned that demographic factors play an important role in vaccine acceptability [6-9]. Khubchandani [6] revealed that in the United States, the highest prevalence of COVID-19 vaccine hesitancy existed in some groups, such as African Americans, Hispanics, and individuals with lower education and incomes. Petravić et al [7] found that in Slovenia, a higher intention to get vaccinated was associated with men, older respondents, physicians, medical students, etc. In addition, some studies applied mining of social media data to perform cross-sectional analysis [10,11]. Hou et al [10] investigated vaccine-related posts from 5 global metropolises between June and July 2020. They discovered that vaccine hesitancy was prevalent worldwide and negative tweets attracted higher engagement on social media. While the cross-sectional method helps us identify diverse attitudes toward vaccines among different demographic groups at a particular time, it cannot track the evolution of attitudes over time.

A longitudinal study is a method that observes some specific variables over an extended period of time. Many studies have applied this method to track trends in population attitudes toward COVID-19 vaccines based on data mining, as data mining can process long-term and large-scale data. Pullan et al [12] and An et al [13] analyzed data from Google Trends, and they both found that the number of searches related to COVID-19 vaccines had increased during the pandemic. Yin et al [14] proposed a novel behavioral dynamics model on Weibo messages to analyze vaccine acceptance in China, and they demonstrated that Chinese individuals were inclined to be positive about side effects over time. Furthermore, many studies explored tweets related to COVID-19 vaccines to understand the evolution of public concerns and sentiments in different regions [15-17]. These studies mainly revealed that public concerns and sentiments on COVID-19 vaccines fluctuated with time and geography, and had strong correlations with some major events about COVID-19 vaccines. Although the longitudinal method applied in the above studies can track the general attitude trend of a population, it cannot identify the disparities of attitudes among demographic groups.

Considering the above-mentioned limitations, we combined cross-sectional and longitudinal analyses in this work to study the online attitudes toward COVID-19 vaccines based on data mining results of tweets. By doing this, we can not only track long-term evolution, but also discern the disparities of attitudes among various population groups. In addition, this work explored the correlation between the whole population’s attentiveness toward vaccines and official COVID-19 statistics, and analyzed the abrupt influences of some major vaccine-related events. These findings can be used as a guide to assist governments and public health organizations in monitoring the trends of different population groups and relieving the low sentiments of specific groups. It is worth mentioning that the method proposed in this study can be easily reutilized to track the attitude evolution of population groups toward any other public health events. The source code developed in this study has been publicly released via GitHub for follow-up research [18].

The remainder of this paper is organized as follows. The Methods section first introduces the data collection and preprocessing procedures, and then presents the structure of a 2-step methodology with its essential design details. The Results section analyzes the mining outcomes from multiple dimensions. Finally, the Discussion section concludes the work.

## Methods

### Data Collection and Preprocessing

The Twitter data used in this study were randomly collected with our self-designed program using the Twitter application programming interface (API) [19] from June 9, 2020, to July 31, 2021, which covered some critical phases concerning the development and inoculation of COVID-19 vaccines. Moreover, the detailed user metadata (such as user name, biography, profile image, user creation time, and follower number) of each tweet were collected simultaneously by setting the Twitter API’s optional query parameters. In total, over 1.4 billion tweets were collected during the research period.

So far, there have been a number of publicly released COVID-19 data sets available for scientific research, such as the data set released by Chen et al [20]. Compared with these public COVID-19 data sets, our data set has the following 2 obvious advantages for our research. First, public COVID-19 data sets only contain tweet IDs in compliance with Twitter’s terms and conditions [21], so extra effort is needed to hydrate all tweet contents from tweet IDs [22]. In contrast, our data set needs less processes since all tweet contents are immediately available without hydration. The second advantage is much more crucial and ultimately led us to decide to use our data set. For public COVID-19 data sets, only up-to-date details on users can be retrieved from user IDs (extracted from hydrated tweet objects) at the time of hydration [23], while our data set already has detailed user metadata at the posting time of the tweets. Usually, user metadata would change more or less over 1 year, so the data at the time of hydration may not be able to infer the demographic characteristics of Twitter users at the posting time of the tweets. Therefore, our data set is more suitable than public COVID-19 data sets for studying the attitudes under various demographic characteristics.

**Figure 2:**
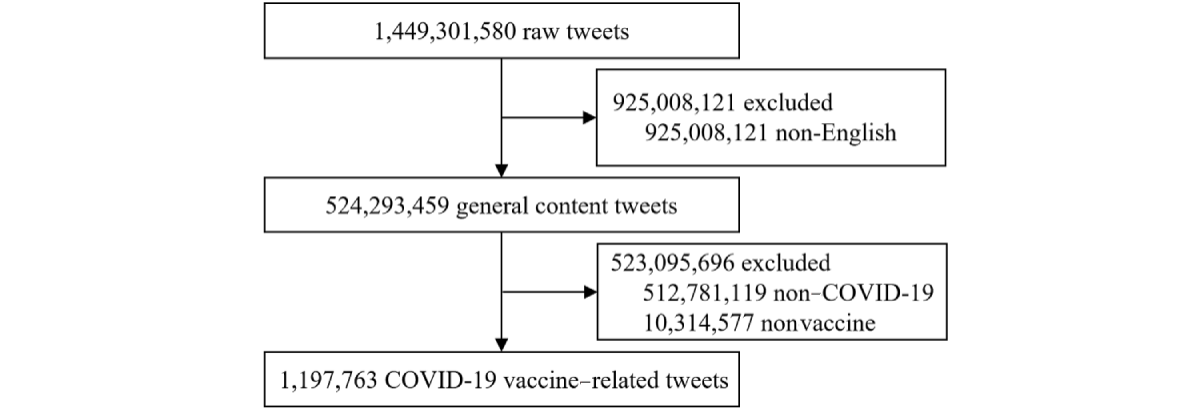
Data selection.

As each tweet in our data set contains a detailed user profile, tweet text, a creation time, a location, statistics, and some other structured data, it can be treated as one online participant with the characteristics of an individual or organization, carrying an attitude for some specific topic. Since English is the most widely spoken language worldwide, we first excluded 925,008,121 non-English tweets by the language attribute of the tweet object, and obtained 524,293,459 English tweets on general content (hereinafter referred to as general content tweets). Then, we excluded 512,781,119 non-COVID-19–related tweets with a filtering pattern composed of 590 COVID-19 keywords and hashtags according to Twitter COVID-19 filtering rules [24]. To concentrate on vaccine-related tweets, we further excluded 10,314,577 non-vaccine tweets. Finally, in this study, we mainly focused on 1,197,763 vaccine-related tweets during COVID-19 (Figure 2) and used general content tweets as a benchmark of general population distribution.

### Study Design

**Figure 3:**
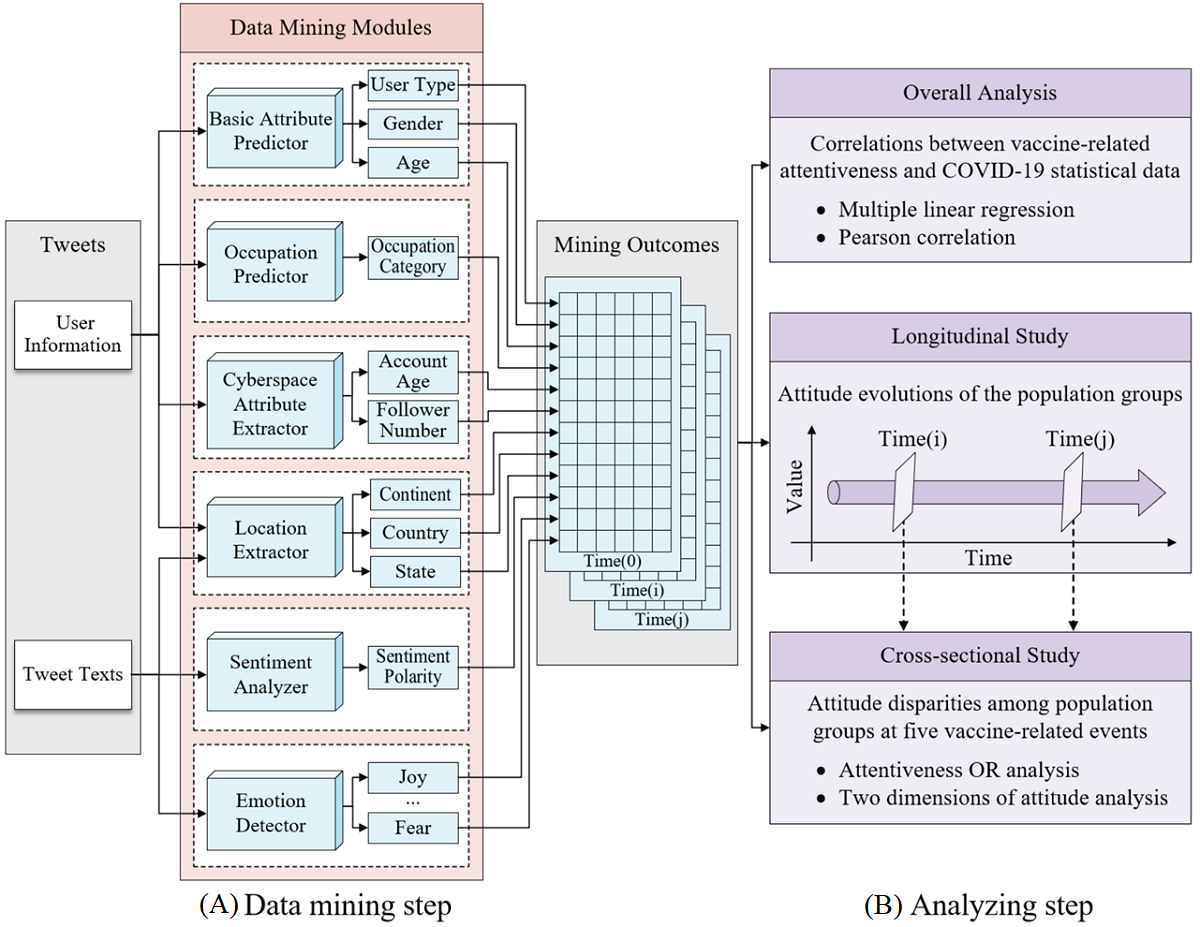
The structure of the 2-step methodology in our study.

We designed a 2-step methodology for this study, as shown in Figure 3. The implementation details of each step are described in the following sections.

### Data Mining Step

The data mining step plays a fundamental and decisive role in the entire research. We applied natural language processing, image processing, and tag extracting algorithms on tweets to extract users’ real-world characteristics (user type, gender, age, occupation, and location), cyberspace characteristics (account age and follower number), sentiment polarities, and emotion types. One set of mining outcomes from a tweet constitutes one record of a user on a specific date. This step is analogous to the process of conventional questionnaire design and result collection. However, the data mining method can flexibly adjust the demographic characteristics that need to be analyzed, and acquire a stable amount of historical data from any population group during a long time period. This step contains 6 intelligent modules, which are described as follows.

#### Basic Attribute Predictor

**Figure 4:**
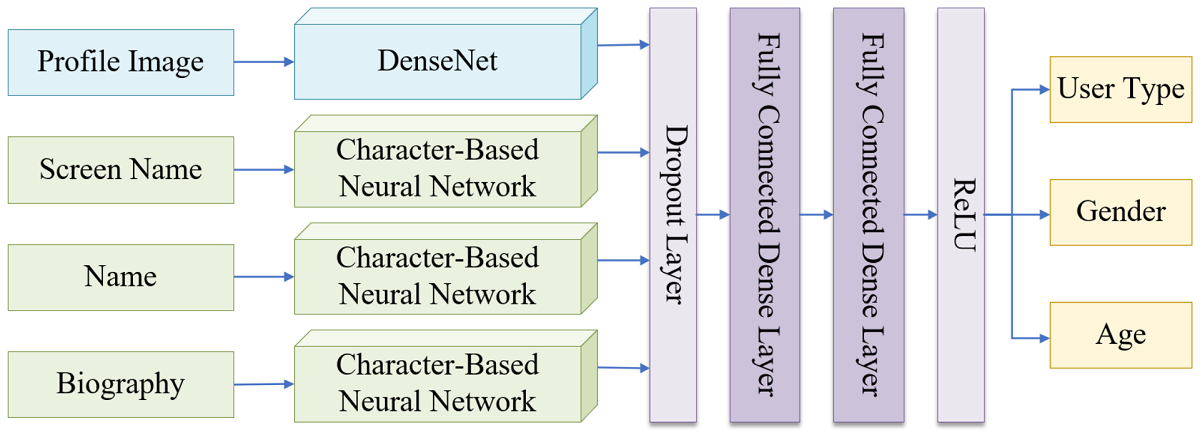
The structure of the M3 (multimodal, multilingual, and multiattribute) model. DenseNet: dense convolutional network; ReLU: rectified linear unit.

This predictor, implemented with an open-source package of the M3 (multimodal, multilingual, and multiattribute) model [25], was employed to predict the probabilities of the following 3 basic demographic attributes: user type, gender, and age, through profile images, screen names, names, and biographies. As shown in Figure 4, the M3 model consists of 1 DenseNet module to process images, 3 character-based neural networks to process text, and finally 2 fully connected dense layers to predict the user type, gender, and age attributes. User type (individual or organization) and gender (male or female) are modeled as binary classification tasks, while age is modeled as a 4-class classification task with the following age groups: ≤18, 19-29, 30-39, and ≥40 years. This model was trained on a massive data set, including Twitter, IMDB, and Wikipedia data [26], and was fine-tuned to capture accurate demographic features. In a previous study, we had tested the M3 model on a subset of our tweet data set, and obtained the benchmark performance as follows: for user type, gender, and age attributes, the accuracy scores were 99.07%, 95.88%, and 77.65%, respectively, and the macro-F1 scores were 0.9860, 0.9572, and 0.7311, respectively [27]. Detailed information about this model can be found in a previous report [25].

#### Occupation Predictor

**Figure 5:**
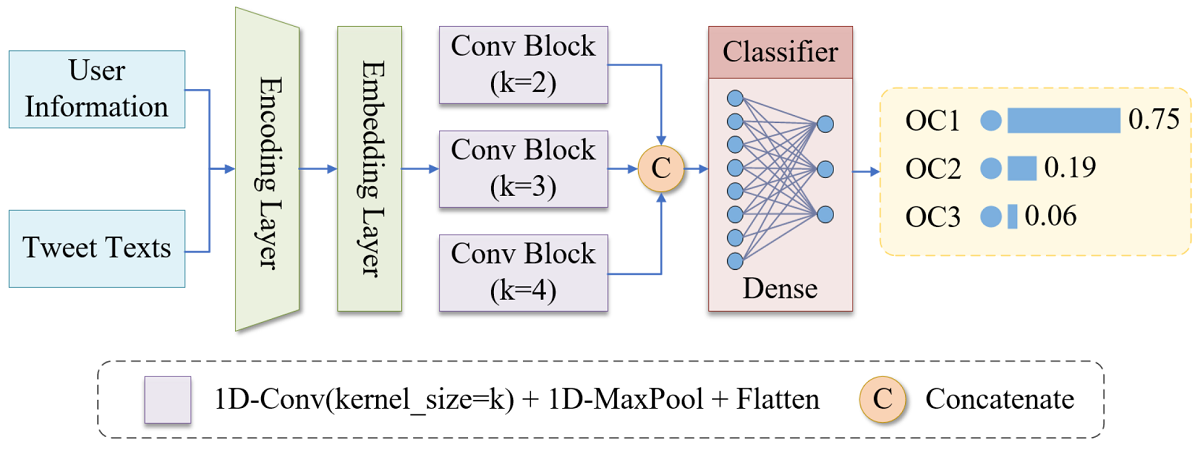
The structure of the occupation predictor. Conv Block: convolution block; 1D-Conv(kernel_size=k): 1D convolution layer with a kernel size of k; 1D-MaxPool: 1D max-pooling layer; Flatten: flatten layer; OCi: the ith occupation category, i∈(1,3).

We constructed a word-based convolutional neural network for occupation inference through user information (such as biographies) and tweet text. As shown in Figure 5, this deep learning model consists of 1 encoding layer, 1 embedding layer, 3 parallel convolution blocks, and finally 1 dense layer to classify the results into 3 classes. Every convolution block is composed of a 1D convolution layer with a different kernel size (eg, 2, 3, and 4), a 1D max-pooling layer, and a flatten layer. The training data we used are from a publicly available data set [28] that has 5191 Twitter users annotated with 9-class occupation categories (OCs), which are defined based on the Standard Occupation Classification (SOC) from the United Kingdom [29]. However, the 9-class classification of Twitter occupation is a challenging task, and the results of the latest studies are not accurate enough for our study. For example, Pan et al [30] employed a 3-layer graph convolutional network (GCN) model to predict the 9-class occupations, and the best performance of accuracy was 61.0%. Therefore, we simplified the 9-class classification into a 3-class classification, and the OCs were abbreviated as OC1, OC2, and OC3. Considering the relationship of the SOC classes and the balance of the 3 categories in the training set, we designed a new occupation division, as shown in Table 1. By adopting 10-fold cross-validation, the accuracy of the occupation predictor reached 74.08% on the new data set.

**Table 1:** The new occupation categories in our study and the original occupation categories in the Standard Occupation Classification hierarchy.

New occupation category (OC)	Original occupation category
OC1	C2: professional occupations
OC2	C1: managers, directors, and senior officials C3: associate professional and technical occupations
OC3	C4: administrative and secretarial occupations C5: skilled trade occupations C6: caring, leisure, and other service occupations C7: sales and customer service occupations C8: process, plant, and machine operatives C9: elementary occupations

#### Cyberspace Attribute Extractor

In this study, we only focused on the following 2 key attributes in cyberspace: account age and follower number. As a matter of fact, there are plenty of cyberspace attributes recorded in the tweet object, such as verified status and tweet number. The reason why we chose these 2 attributes is that account age can reflect the internet age of a user, and follower number can indicate a user’s influence and usage level of social media to a certain extent. Additionally, Lyu et al [31] studied the characterization of population groups with varied attitudes toward COVID-19 vaccines and concluded that account age and follower number also have an influence on population attitudes toward vaccines. The account age of a user was calculated by subtracting the user’s creation time from the tweet’s creation time, and the follower number was retrieved directly from the “stats” field of the tweet object.

#### Location Extractor

We used the “geo” field in the tweet object and the “location” field in the profile of a Twitter user to efficiently extract location information, including the continent, country, and state. The extraction process was implemented in 2 steps. First, the location extractor called the Twitter API to query a place by the geocode in the “geo” field [32] and then obtained the exact location from the retrieved data. Second, if a tweet did not contain a geocode, the extractor used the “location” field to fuzzy inquire the location, which was implemented by GeoPy [33] and pycountry [34]. Through these 2 steps, approximately 63.41% of Twitter users’ locations could be extracted.

#### Sentiment Analyzer

The sentiment analyzer was implemented with an open-source tool named Valence Aware Dictionary and Emotional Reasoner (VADER) [35]. It is a lexicon and rule-based sentiment analysis tool specifically attuned to sentiments expressed in social media. In this study, we used VADER to calculate the sentiment polarities (−1 to 1) of the tweet text and then divided the results into the following 3 subranges: negative (–1 to –0.05), neutral (–0.05 to 0.05), and positive (0.05 to 1).

#### Emotion Detector

The emotion detector was based on an open-source emotion recognition algorithm [36] on Twitter that utilized a character-based trained recurrent neural network algorithm. In the original study, it implemented 3 different emotion models. We selected the model of Ekman’s 6 basic emotions [37] (anger, disgust, fear, joy, sadness, and surprise) to predict the emotion types of the tweets in this study.

### Analyzing Step

Based on the mining outcomes, we conducted multiple analyses, including overall analysis, a longitudinal study, and a cross-sectional study, to detect the evolution and disparities of attitudes toward COVID-19 vaccines among population groups during the study period. The multiple analyses are shown in Figure 3.

Concretely, in the overall analysis, multiple linear regression and Pearson correlation analysis were used to detect the impacts of official COVID-19 statistics, including confirmed cases, deaths, vaccinations, and reproduction rate, and some major vaccine-related events on the whole population’s attentiveness toward vaccines. In the longitudinal study, to detect the evolution of attitudes among different population groups over time, a series of longitudinal contrasts with different demographic characteristics were displayed and analyzed. To further reveal the attitude patterns among population groups, a cross-sectional study, incorporating the benchmark of the population distribution of the general content tweets, was conducted at 5 vaccine-related events selected from the overall analysis.

The strength of the correlation and similarity in this paper using the guide that Evans [38] suggested for the absolute value of Pearson *r* can be described as follows: very weak (0 to 0.19), weak (0.20 to 0.39), moderate (0.40 to 0.59), strong (0.60 to 0.79), and very strong (0.80 to 1).

## Results

### Overall Analysis

In this section, we explore the possible influencing factors of the whole population’s attentiveness toward vaccines in 2 steps. First, studying the correlation between attentiveness and official COVID-19 statistics. Second, discovering the abrupt influences of some major vaccine-related events during COVID-19. At the end of this section, the data mining results of vaccine-related tweets and general content tweets have been displayed and analyzed in general.

#### The Influencing Factors of the Attentiveness Toward Vaccines

**Figure 6:**
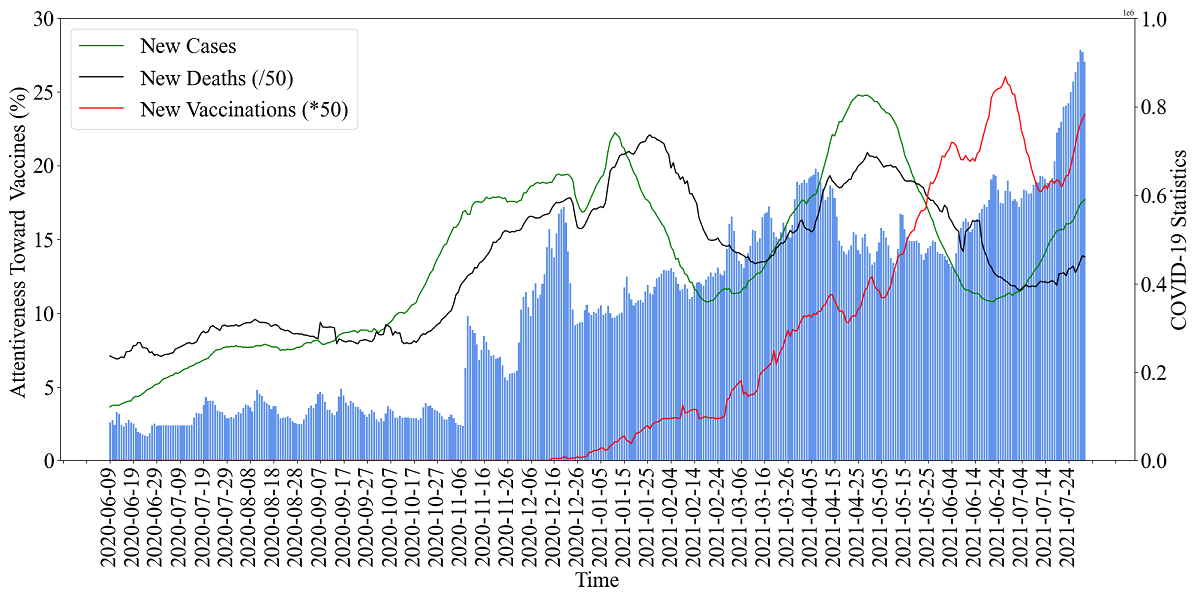
The whole population’s attentiveness toward vaccines, and the COVID-19 statistics during the study period. The y-axis on the left is the level of attentiveness, and the y-axis on the right represents the numbers of COVID-19 statistics, which adopt different scales.

In order to eliminate possible fluctuations in the quantities of tweets captured daily, we used the percentage of vaccine-related tweets in COVID-19 tweets to represent the attentiveness toward vaccines (sometimes referred to as attentiveness for short in the following text) during COVID-19 in this study. Meanwhile, we obtained the global data of COVID-19 statistics from Our World in Data [39], including the daily numbers of new cases, new deaths, new vaccinations, total cases, total deaths, total vaccinations, and people vaccinated, as well as the reproduction rate. Three of the statistics are plotted as examples in Figure 6.

Since some variables of the COVID-19 statistics do not exert an immediate influence on the attentiveness toward vaccines, we applied Pearson correlation analysis on different time delays (0≤lag≤30 days) of attentiveness with each of the statistical variables, and found that the optimal lag at which Pearson *r* reached the absolute maximum value was different for each variable, as shown in Table 2. Moreover, all variables of the COVID-19 statistics showed positive correlations with attentiveness, except for reproduction rate, which showed a negative correlation, with *r*=−0.4562 at lag=10.

**Table 2:** Pearson correlation coefficients between COVID-19 statistics and attentiveness toward vaccines with lags that have absolute maximum r values within 30 days.

Variable	Lag days	Pearson *r*	*P* value
New cases	5	0.5917	<.001
New deaths	3	0.6543	<.001
New vaccinations	4	0.7843	<.001
Total cases	0	0.9093	<.001
Total deaths	0	0.9066	<.001
Total vaccinations	0	0.7433	<.001
People vaccinated	0	0.7364	<.001
Reproduction rate	10	−0.4562	<.001

Then, we took the 8 variables of COVID-19 statistics as independent variables (denoted as X*_i_*(*t*), *i*∈(1,8) and X*_0_*(*t*)=1, so X(*t*) is a 9-dimensional vector) and attentiveness as a dependent variable (denoted as Y(*t*)). Multiple linear regression was used to analyze the relationship between X(*t*) and Y(*t*), which is expressed as follows:




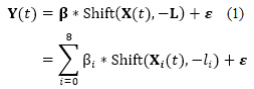




In this formula, **β** is a coefficient vector of the shifted independent variables. Shift is a function to make a shift −L on X(*t*). **L** is a shift vector composed of the lag values taken from Table 2, that is, L=(0,5,3,4,0,0,0,0,10). Since the shifting direction of these lags is for attentiveness, when shifting the independent variables X(*t*), we take the opposite direction of **L**, that is −**L**.

**Figure 7:**
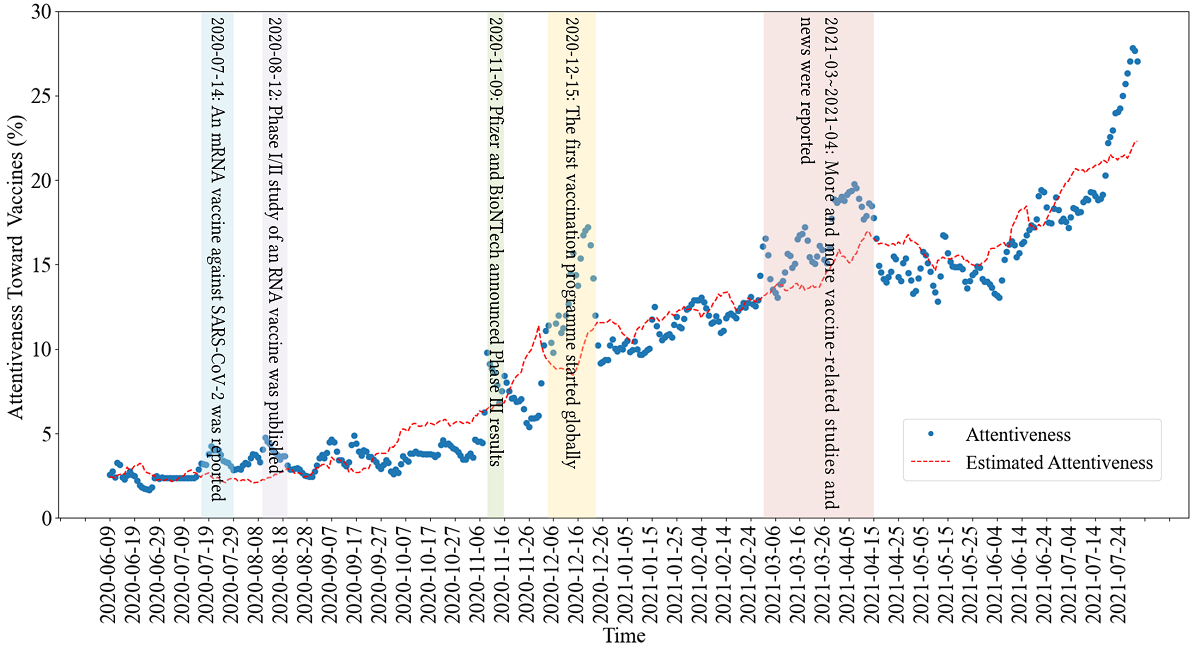
The whole population’s attentiveness toward vaccines, estimated attentiveness by COVID-19 statistics, and labels of 5 major vaccine-related events.

By using the above regression model between the attentiveness Y(*t*) and the COVID-19 statistical vector X(*t*), we obtained the coefficient vector β, the adjusted R-squared value, which reached 0.9034, and the estimated attentiveness Ŷ(*t*). The Pearson correlation between Y(*t*) and Ŷ(*t*) was 0.9512 (*P*<.001), which was higher than that for Y(*t*) with any single independent variable X*_i_*(*t*) in Table 2. Therefore, the whole population’s attentiveness toward vaccines and the estimated attentiveness by COVID-19 statistics showed a very strong positive correlation. The curves of the attentiveness Y(*t*) and estimated attentiveness Ŷ(*t*) are shown in Figure 7.

Furthermore, we noticed that some abrupt jumps appeared in the attentiveness curve. An earlier study by Chen et al [20] concluded that Twitter discourse statistics can reflect major events at the time. Inspired by this, we calculated the periods when the attentiveness was significantly higher than the fitted curve and lasted more than 5 days. We used the standard deviation of the gaps between daily attentiveness and the fitted data as a threshold for judging the significant level. After that, we found that these periods exactly corresponded to some major vaccine-related events, as shown in Table 3. Compared to the development process of COVID-19 vaccines, the time points of these vaccine-related events can roughly fall into the following phases: *t*_1_, *t*_2_, and *t*_3_ in phase I/II/III of the vaccine clinical trial period, and *t*_4_ and *t*_5_ in the vaccination period. Since each manufacturer has its own schedule for developing COVID-19 vaccines, the description of the vaccine phases here is used only as a reference for this study.

**Table 3:** Some major events related to COVID-19 vaccines.

Time	Major event	Highest attentiveness percentage	Lasting days
July 14, 2020 (*t*_1_)	“An mRNA vaccine against SARS-CoV-2—preliminary report” [40] was published.	4.27%	14
August 12, 2020 (*t*_2_)	“Phase I/II study of COVID-19 RNA vaccine BNT162b1 in adults” [41] was published.	4.78%	12
November 9, 2020 (*t*_3_)	Pfizer and BioNTech announced phase III results [42].	9.78%	6
December 15, 2020 (*t*_4_)	The first mass vaccination program started globally [43].	17.48%	21
April 10, 2021 (*t*_5_) selected from March to April, 2021	More and more vaccine-related studies and news were reported (see Multimedia Appendix 1).	18.92%	46

The above data analysis shows that the total online population’s attentiveness toward COVID-19 vaccines was significantly correlated with COVID-19 statistics, including confirmed cases, deaths, vaccinations, and reproduction rate. Besides, attentiveness was also influenced by some vaccine-related events.

#### Statistics of Inferred Latent Characteristics

Two data mining experiments were conducted on 2 types of tweets as a comparison to infer different latent characteristics. The data mining methods are described in the Data Mining Step of the Methods section. The first experiment was performed on 1,197,763 vaccine-related tweets covering 1 year, and the second experiment was on 100,000 general content tweets selected from 5 major vaccine-related events (20,000 samples at each event) described in Table 3. The purpose of the second experiment was to get a benchmark of the general population distribution. The overall statistics of the 2 types of tweets are summarized and shown in Table 4. *P* values are calculated by the chi-square test.

**Table 4:** Overall statistics of vaccine-related tweets and general content tweets.

Characteristic		Vaccine-related tweets (N=1,197,763)	General content tweets at 5 major time points (N=100,000)	*P* value
**User type, n (%)**				<.001
	Individual	1,079,105 (90.09)	94,560 (94.56)	
	Organization	118,658 (9.91)	5440 (5.44)	
**Gender, n (%)**				<.001
	Male	661,511 (61.30)	50,156 (53.04)	
	Female	417,594 (38.70)	44,404 (46.96)	
**Age (years), n (%)**				<.001
	≤18	157,395 (14.59)	35,036 (37.05)	
	19-29	254,920 (23.62)	32,142 (33.99)	
	30-39	202,451 (18.76)	11,112 (11.75)	
	≥40	464,339 (43.03)	16,270 (17.21)	
**Occupation category (OC), n (%)**				<.001
	OC1	385,276 (35.70)	20,102 (21.26)	
	OC2	347,257 (32.18)	31,176 (32.97)	
	OC3	346,572 (32.12)	43,282 (45.77)	
**Location–continent^a^, n (%)**				<.001
	North America	274,565 (40.12)	21,202 (37.45)	
	Europe	179,683 (26.26)	13,132 (23.20)	
	Asia	106,713 (15.60)	12,388 (21.88)	
	Africa	67,805 (9.91)	7320 (12.93)	
	Oceania	39,414 (5.76)	1250 (2.21)	
	South America	15,794 (2.31)	1292 (2.28)	
	Antarctica	301 (0.04)	26 (0.05)	
**Location–country^a,b^, n (%)**				<.001
	United States	214,606 (31.36)	18,163 (32.09)	
	United Kingdom	106,337 (15.54)	6334 (11.19)	
	India	51,698 (7.56)	2969 (5.24)	
	Canada	42,554 (6.22)	1538 (2.72)	
	Australia	20,477 (2.99)	474 (0.84)	
**Account age (years), n (%)**				<.001
	<5	478,914 (44.38)	58,341 (61.70)	
	5-10	349,595 (32.40)	26,333 (27.85)	
	≥10	250,596 (23.22)	9886 (10.45)	
**Follower number, n (%)**				<.001
	<500	619,808 (57.44)	55,745 (58.95)	
	500-5000	368,780 (34.17)	32,742 (34.63)	
	≥5000	90,517 (8.39)	6073 (6.42)	
**Sentiment polarity**				<.001
	Overall (–1 to 1), mean (SD)	0.0161 (0.4591)	0.1215 (0.4566)	<.001
	Negative (–1 to −0.05), n (%)	411,990 (38.18)	41,555 (43.95)	
	Neutral (–0.05 to 0.05), n (%)	292,273 (27.08)	29,987 (31.71)	
	Positive (0.05 to 1), n (%)	374,842 (34.74)	23,018 (24.34)	
**Emotion, n (%)**				<.001
	Fear	528,667 (48.99)	21,703 (22.95)	
	Joy	313,423 (29.04)	31,819 (33.65)	
	Surprise	153,807 (14.25)	28,690 (30.34)	
	Sadness	42,124 (3.90)	8940 (9.45)	
	Anger	23,814 (2.21)	2570 (2.72)	
	Disgust	17,270 (1.60)	838 (0.89)	

^a^Under location (continent and country) characteristics, the total number of vaccine-related tweets with location information was 684,275, and the total number of general content tweets with location information was 56,610.

^b^The top 5 countries with the most vaccine-related tweets are selected.

From Table 4, it can be seen that there were significant differences (*P*<.001) between vaccine-related tweets and general content tweets. For example, the proportions for males and females were 53.04% and 46.96% in general content tweets, which were relatively balanced in gender. However, the gender gap enlarged in vaccine-related tweets to 22.60% (61.30% for males and 38.70% for females). The top 3 emotions in general content tweets were *joy* (33.65%), *surprise* (30.34%), and *fear* (22.95%), while in vaccine-related tweets, the top 3 were *fear* (48.99%), *joy* (29.04%), and *surprise* (14.25%).

In the next 2 sections, we further analyzed these long-term and multicharacteristic data in longitudinal and cross-sectional studies.

### Longitudinal Study

In this section, we analyzed the attitude evolution of population groups from the following 2 aspects: attentiveness and sentiments toward vaccines.

#### Attentiveness Toward Vaccines Among Different Population Groups

**Figure 8:**
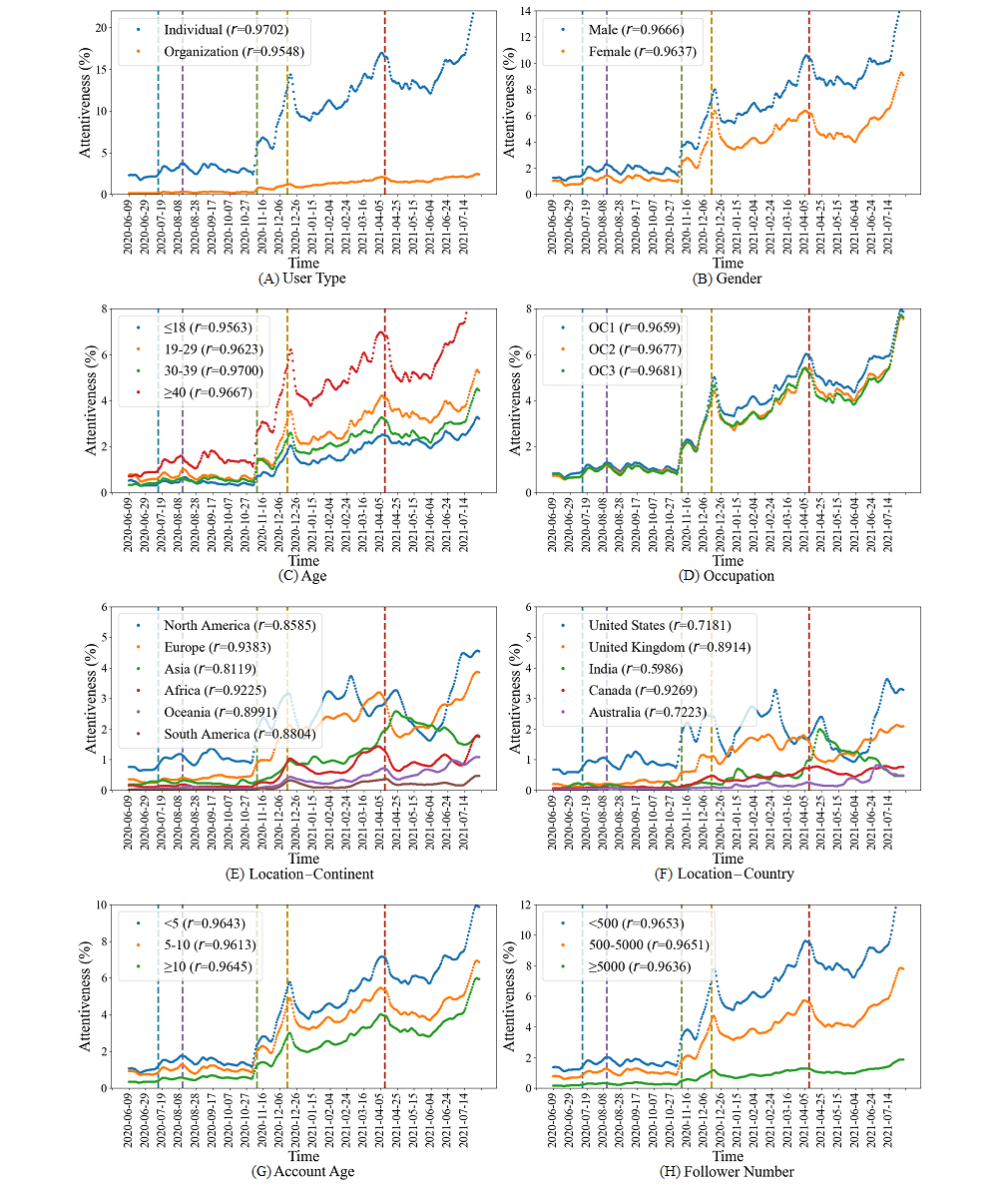
Attentiveness toward vaccines of different population groups. The vertical dashed lines represent the time points of 5 major vaccine-related events as follows: July 14, 2020 (*t*1), August 12, 2020 (*t*2), November 9, 2020 (*t*3), December 15, 2020 (*t*4), and April 10, 2021 (*t*5). The *r* values in the legend boxes are the Pearson correlation coefficients between each population group and the whole population. OC: occupation category.

Based on the data mining outcomes, we obtained the daily attentiveness toward vaccines of population groups by calculating the percentages of vaccine-related tweets in COVID-19 tweets with different characteristics. As shown in Figure 8, in general, the attentiveness of each group increased over time. At the 5 major vaccine-related events, most of the population groups had local peaks, similar to the whole population’s attentiveness. By calculating the Pearson *r* values, it was shown that the attentiveness of all population groups had very strong similarities (*r*>0.80) with that of the whole population, except for the United States and Australia that had strong similarities (0.60<*r*<0.79) and India that had a moderate similarity (0.40<*r*<0.59).

Furthermore, except for 2 location characteristics, population groups under the 6 demographic characteristics exhibited consistent differences throughout the study period. For example, males always had a higher level of attentiveness than females. In contrast, there was no specific pattern for population groups under the 2 location characteristics. In particular, North America almost had the highest attentiveness among all continents during the pandemic, while Europe and Asia surpassed it in some periods. Moreover, the United States almost had the highest attentiveness among all countries, while the United Kingdom and India surpassed it in some periods.

In summary, most of the population groups had very strong similarities with the whole population regarding attentiveness, that is, attentiveness increased over time with some local peaks, indicating that they might be affected by the vaccine-related events as well. Moreover, there existed consistent group differences in the evolution of attentiveness under the demographic characteristics, except for the 2 location characteristics.

#### Sentiments Toward Vaccines Among Different Population Groups

**Figure 9:**
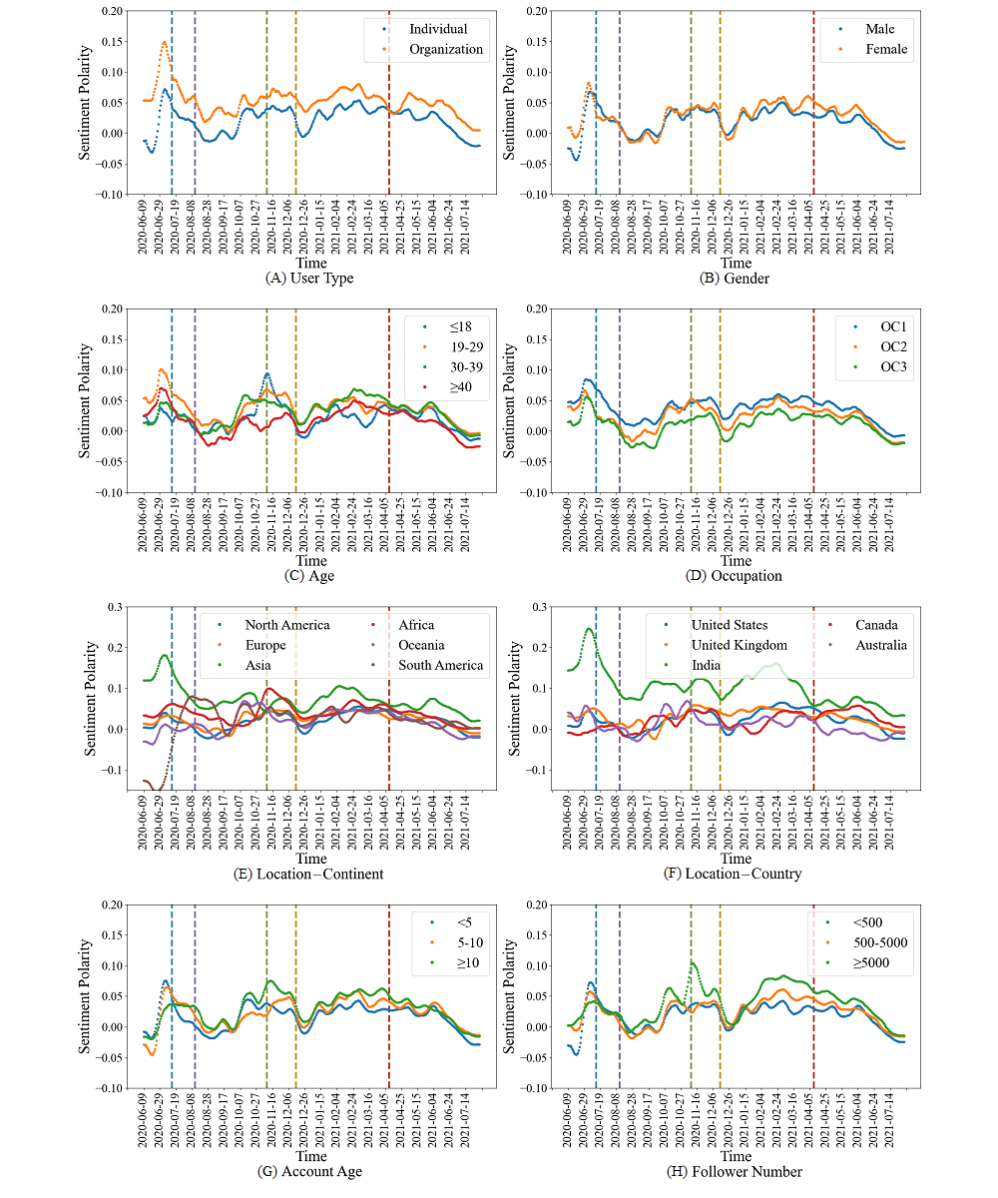
Sentiments toward vaccines of different population groups. OC: occupation category.

As shown in Figure 9, we calculated the daily mean sentiment polarities of the population groups under the demographic characteristics. In general, the sentiments of all population groups fluctuated greatly in the early period of the vaccine development, and gradually stabilized at the start of vaccination (*t*_4_). Nevertheless, they all went down after June 2021.

In particular, the sentiments of organizations were more positive than individuals. Females were sometimes a bit less positive than males in the development phase of vaccines, but were more positive than males after the inoculation started. Among the 4 age groups, the sentiments of people aged ≥40 and ≤18 years were almost the lowest during the study period. Among the 3 categories of occupations, the sentiments of OC1 were the highest, while those of OC3 were always the lowest. Under the 2 location characteristics, South America exhibited a different sentiment trend compared with other continents. Asia among the continents and India among the countries had the highest sentiments, and both showed downward trends. Among the 3 account age groups, the group of <5 years almost had the lowest sentiments. Among the follower number groups, the group of <500 followers nearly had the lowest sentiments, while the group of ≥5000 followers had the highest sentiments, except for the period before *t*_2_.

In summary, the sentiments differed among population groups and fluctuated a lot in the early period of vaccine development, which suggested that different populations might hold different and immature views at the beginning. After June 2021, there were downward trends in all populations, indicating that populations might become less positive toward vaccines than before.

### Cross-sectional Study

In the previous section, we mainly focused on the long-term evolution of population attitudes toward vaccines among COVID-19–related tweets, ignoring the general population distribution. Actually, the sizes of population groups vary greatly with respect to population characteristics. Thus, it is meaningful and essential to investigate the attentiveness ratios toward vaccines among different population groups under the benchmark of the general population distribution. Therefore, in this section, we conducted 5 cross-sectional analyses at 5 major vaccine-related events, by applying the odds ratio (OR) to represent the attentiveness ratio toward vaccines of each population group. Due to the complexity of attentiveness among continents and countries under the 2 location characteristics, we only analyzed the 6 demographic characteristics.

As shown in Table 5, considering the general population distribution, the attentiveness ratios toward vaccines of organizations were higher than that of individuals at all the time points, with the OR ranging from 1.44 (95% CI 1.28-1.61) to 2.01 (95% CI 1.70-2.39). The attentiveness ratios of females were lower than that of males, with the OR ranging from 0.61 (95% CI 0.57-0.66) to 0.82 (95% CI 0.77-0.88). Under the age characteristic, the OR increased progressively with age at all 5 time points. The ≥40 age group always had the highest attentiveness ratios, with the OR ranging from 3.70 (95% CI 3.53-3.87) to 8.81 (95% CI 8.53-9.10). Under the occupation characteristic, OC3’s attentiveness ratios were the lowest among the 3 categories of occupations, with the OR ranging from 0.37 (95% CI 0.30-0.44) to 0.44 (95% CI 0.39-0.49). The attentiveness ratios of the account age groups showed the same trends as the real-world age groups, with the attentiveness OR increasing with account age. Under the follower number characteristic, a higher number of followers was associated with a higher attentiveness OR.

**Table 5:** Attentiveness odds ratios toward vaccines at 5 major vaccine-related events.

Characteristic			Major vaccine-related events								
			*t*_1_: July 14, 2020, attentiveness OR^a^ (95% CI)		*t*_2_: August 12, 2020, attentiveness OR (95% CI)		*t*_3_: November 9, 2020, attentiveness OR (95% CI)		*t*_4_: December 15, 2020, attentiveness OR (95% CI)		*t*_5_: April 10, 2021, attentiveness OR (95% CI)
**User type**											
	Individual	1 (ref^b^)		1 (ref)		1 (ref)		1 (ref)		1 (ref)	
	Organization	1.51 (1.20-1.89)		1.46 (1.22-1.76)		2.01 (1.70-2.39)		1.44 (1.28-1.61)		1.98 (1.79-2.19)	
**Gender**											
	Male	1 (ref)		1 (ref)		1 (ref)		1 (ref)		1 (ref)	
	Female	0.75 (0.66-0.86)		0.79 (0.71-0.87)		0.66 (0.60-0.73)		0.82 (0.77-0.88)		0.61 (0.57-0.66)	
**Age (years)**											
	≤18	1 (ref)		1 (ref)		1 (ref)		1 (ref)		1 (ref)	
	19-29	1.68 (1.55-1.82)		1.49 (1.42-1.56)		1.85 (1.78-1.93)		2.31 (2.23-2.39)		2.13 (2.05-2.20)	
	30-39	3.55 (3.26-3.86)		2.31 (2.19-2.44)		4.48 (4.30-4.66)		4.39 (4.23-4.55)		5.22 (5.03-5.42)	
	≥40	5.26 (4.89-5.67)		3.70 (3.53-3.87)		7.34 (7.08-7.60)		7.12 (6.90-7.35)		8.81 (8.53-9.10)	
**Occupation category (OC)**											
	OC1	1 (ref)		1 (ref)		1 (ref)		1 (ref)		1 (ref)	
	OC2	0.52 (0.44-0.63)		0.62 (0.55-0.70)		0.66 (0.61-0.72)		0.52 (0.48-0.57)		0.59 (0.55-0.64)	
	OC3	0.37 (0.30-0.44)		0.44 (0.39-0.49)		0.42 (0.39-0.46)		0.41 (0.38-0.45)		0.42 (0.39-0.45)	
**Account age (years)**											
	<5	1 (ref)		1 (ref)		1 (ref)		1 (ref)		1 (ref)	
	5-10	1.31 (1.10-1.55)		1.34 (1.20-1.49)		1.67 (1.54-1.81)		2.28 (2.12-2.46)		2.25 (2.08-2.44)	
	≥10	1.93 (1.54-2.40)		1.62 (1.40-1.87)		2.88 (2.62-3.17)		3.71 (3.40-4.04)		3.28 (3.01-3.58)	
**Follower number**											
	<500	1 (ref)		1 (ref)		1 (ref)		1 (ref)		1 (ref)	
	500-5000	0.97 (0.82-1.14)		1.02 (0.92-1.13)		1.04 (0.96-1.13)		1.07 (0.99-1.14)		1.02 (0.95-1.10)	
	≥5000	1.32 (1.00-1.74)		1.20 (1.00-1.44)		1.69 (1.48-1.92)		1.43 (1.27-1.61)		1.40 (1.23-1.59)	

^a^OR: odds ratio.

^b^ref: reference.

**Figure 10:**
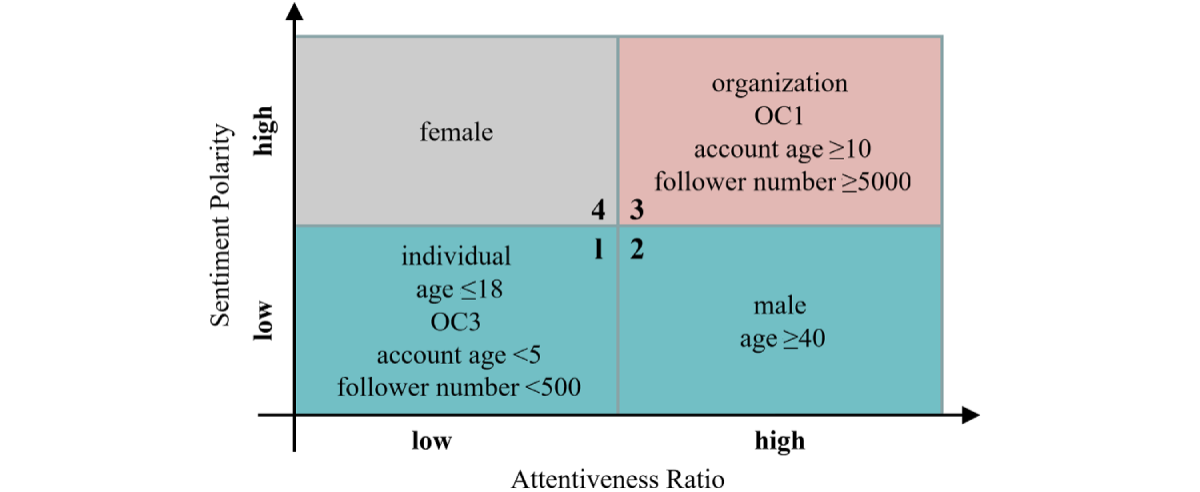
Four categories of population groups by crossing the 2 dimensions of attitude toward vaccines. OC: occupation category.

To further discern the deep law of attitudes under different demographic characteristics after the inoculation started, we crossed the 2 dimensions of attitude (the attentiveness ratio and sentiment polarity) according to the cross-sectional results at *t*_4_ and *t*_5_. The levels of the attentiveness ratios and sentiment polarities can be found in Table 5 and Figure 9 (see Multimedia Appendix 2 for the specific sentiment polarities), respectively. Then, all the population groups were divided into 4 categories, as shown in Figure 10. Each grid in this figure is 1 category combining high or low levels of the 2 dimensions. For example, the 1st category is a combination of low attentiveness ratio and low sentiment polarity, and it includes individuals with age ≤18 years, OC3 (occupations of the 3rd category), account age <5 years, and follower number <500.

From Figure 10, it can be seen that the 1st category and 2nd category both had low sentiments toward vaccines, with either low or high levels of attentiveness ratios. The 1st category was mainly composed of people with relatively low levels of education, low awareness of news, low income, and light use of social media, while the 2nd category was composed of males and older people. The 3rd category had high sentiments and high attentiveness ratios, indicating that these population groups had persistent attention and trust in the COVID-19 vaccines. Some groups in the 3rd category were groups with relatively high levels of social influence, both in reality (organizations) and in cyberspace (account age ≥10 years, follower number ≥5000). These findings can provide comprehensive guidance for governments and public health organizations in deciding who should be given more attention and targeted help, and who can be considered to promote the publicity of vaccines. In the Discussion section, we will further investigate the possible reasons for the categories carrying low sentiments, and provide some practical suggestions for interventions accordingly.

## Discussion

### Principal Findings

In this study, we acquired and analyzed a year-long collection of tweets, from June 9, 2020, to July 31, 2021, to discover the evolution and disparities of attitudes toward COVID-19 vaccines among various online population groups. Overall, the whole population’s attentiveness toward COVID-19 vaccines increased over time with some local fluctuations during the study period. This study demonstrated that this attentiveness had a very strong correlation (Pearson *r*=0.9512) with official COVID-19 statistics, such as confirmed cases and deaths, and it was noticeably influenced by some major vaccine-related events. Studies conducted on other online platforms, such as Google and Baidu, also had results similar to ours. For example, Hu et al [44] found that there was a correlation between the Google Trends of relative search volume for COVID-19 and the daily number of new cases. Besides, by comparing demographic composition and sentiments between vaccine-related tweets and general content tweets, we found that there were significant differences (*P*<.001). In particular, the whole population had lower sentiments and more *fear* toward vaccines than general topics.

By analyzing the attentiveness evolution toward vaccines under 8 demographic characteristics, we observed that, except for the United States, Australia, and India, all population groups exhibited very strong similarities with the whole population. As for the sentiment evolution toward vaccines, we found that different populations initially held different and fluctuated sentiments in the early stage of vaccine development, and then, the sentiments gradually stabilized and tended to relatively positive levels at the beginning of vaccination, but after June 2021, they all had a considerably downward trend. The research findings of Yan et al [45] and Hu et al [46] on sentiment toward COVID-19 vaccines are partially consistent with ours. However, their studies do not have data from June to July 2021 to verify the downward trend in sentiment discovered in our study. By reading news reports during this period, we noticed some possible clues for this trend. The most likely one is the spread of the Delta variant, which is more contagious than the other coronavirus strains [47]. It is claimed that vaccination is still the best protection against the Delta variant. Thus, the downward trend in sentiment is a critical warning signal for governments and public health agencies, calling for more attention dedicated to public concerns regarding COVID-19 vaccines.

Furthermore, there are significant attitude disparities toward COVID-19 vaccines across population groups. By crossing the 2 dimensions of attitude (the attentiveness ratio and sentiment polarity), we found that among population groups carrying low sentiments, some have low attentiveness ratios (the 1st category in Figure 10), such as individuals with age ≤18 years, occupations of the 3rd category, account age <5 years, and follower number <500, while some have high attentiveness ratios (the 2nd category in Figure 10), such as males and individuals with age ≥40 years.

We investigated and inferred the internal reasons for the low sentiments, and found some corresponding epidemiological studies that can confirm our inference. For the 1st category of the population, the low sentiments may be derived from the insufficient knowledge and distrust of vaccines based on education status, news awareness, economic conditions, level of social media usage, etc. This finding appears consistent with the finding of Paul et al [48], who revealed that distrustful attitudes toward vaccination were higher among individuals with lower levels of education, lower annual income, poor knowledge of COVID-19, and poor compliance with government COVID-19 guidelines. The 2nd category of the population may be aware of the high risks of COVID-19 related to their own physical conditions, so they are prone to incur negative sentiments. Medical evidence indicates that males [49] compared to females and older people [50] compared to younger people might have higher morbidity and mortality from COVID-19–related diseases, and they are more willing to be vaccinated [51].

Overall, only paying excess attention blindly cannot effectively allay the diverse public concerns and fears about vaccines. Specialized interventions should be implemented to address these concerns raised by different populations. Some studies in the field of public health are worthy of reference. For example, Brooke et al [52] discussed the problems encountered by older people during COVID-19 and put forward some practical suggestions for older people with comprehensive health help, both psychological and physical in COVID-19, the development of social network communications through online technologies (such as Facebook, Twitter, WhatsApp, and other similar platforms), and some creative ways of virtual entertainment (such as performances of symphony orchestras in virtual concert halls). Malik et al [51] pointed out that to build confidence in COVID-19 vaccines, thoughtful and targeted messaging and education need to be developed, not only for the general American population, but also specifically for high-risk groups. According to this, fantastic short videos or simple messages related to vaccines could be recommended to population groups with low sentiments toward vaccines to arouse their interest and educate them about the safety and effectiveness of vaccines. Additionally, we found that organizations, individuals with occupations of the 1st category, those with account age ≥10 years, and those with follower number ≥5000 were more concentrated and positive toward COVID-19 vaccines, as shown in Figure 10. Inspired by this, we further suggest that public health authorities cooperate with these users by using their social influence to expand the publicity of vaccines.

### Limitations

Due to the high complexity of multilingual analysis and the insufficient support for detecting the various characteristics of the population groups, this paper only extracted data in English and key demographic characteristics for analysis. In addition, considering that too many OCs may reduce the accuracy of the occupation predictor, we only divided the occupations into 3 categories, which may have resulted in the loss of some fine-grained information. Despite these limitations, it did not affect the overall findings.

### Conclusions

By analyzing large-scale tweets during vaccine development and vaccination, this study tracked the year-long evolution of attitudes toward COVID-19 vaccines among population groups, and offered rich evidence to gain insights about the attitude patterns of the international population on social media. Through well-organized approaches, governments, public health agencies, health care providers, and influential Twitter users can work together to help those populations with low sentiments get through this difficult period. At last, it is worth mentioning that the method applied in this paper can be easily extended to other public health events for multidimensional and large-scale research on the long-term evolution of human responses. The source code developed in this study is available for use at GitHub [18].

## Appendix

Multimedia Appendix 1 Supplemental information about vaccine-related news between March and April 2021.

Multimedia Appendix 2 Supplemental information about the sentiments toward vaccines at 5 major vaccine-related events.

## Abbreviations

API: application programming interface
M3: multimodal, multilingual, and multiattribute
OC: occupation category
OR: odds ratio
SOC: Standard Occupation Classification
VADER: Valence Aware Dictionary and Emotional Reasoner
