# Supplementary material for: The Evolution and Disparities of Online Attitudes Toward COVID-19 Vaccines: Year-long Longitudinal and Cross-sectional Study
Source: J Med Internet Res. 2022 Jan 21;24(1):e32394. doi: 10.2196/32394 (PMC8786033; doi:10.2196/32394)
Supplement: Multimedia Appendix 1 [file jmir_v24i1e32394_app1.docx]

**Multimedia Appendix 1.** Supplemental information about vaccine-related news between March and April 2021.

- 2021-03-03: Biden says U.S. will have enough coronavirus vaccine doses for every adult by end of May. URL: <https://www.nbcnews.com/politics/white-house/biden-announce-merck-will-help-manufacture-johnson-johnson-s-coronavirus-n1259262>
- 2021-03-10: Biden orders 100 million more vaccine shots from Johnson & Johnson. URL: <https://www.washingtonpost.com/business/2021/03/10/vaccine-biden-johnson-johnson/>?
- 2021-03-11: Fact Sheet: President Biden to Announce All Americans to be Eligible for Vaccinations by May 1, Puts the Nation on a Path to Get Closer to Normal by July 4th. URL: <https://www.whitehouse.gov/briefing-room/statements-releases/2021/03/11/fact-sheet-president-biden-to-announce-all-americans-to-be-eligible-for-vaccinations-by-may-1-puts-the-nation-on-a-path-to-get-closer-to-normal-by-july-4th/>
- 2021-03-11: European Countries Suspend Use of AstraZeneca Shots Over Worries About Blood Clots. URL: <https://www.nytimes.com/2021/03/11/business/astrazeneca-vaccine-denmark-blood-clots.html>?
- 2021-03-11: 49 percent of GOP men say they won't get vaccinated: PBS poll. URL: <https://thehill.com/policy/healthcare/542814-49-percent-of-gop-men-say-they-wont-get-vaccinated-pbs-poll>
- 2021-03-15: The White House is set to unveil a wide-reaching, billion-dollar campaign aimed at convincing every American to get vaccinated. URL: <https://www.statnews.com/2021/03/15/white-house-unveil-a-wide-reaching-billion-dollar-campaign-convincing-every-american-to-get-vaccinated/>
- 2021-03-15: Germany, France, Spain and Italy become latest countries to suspend AstraZeneca vaccine over blood clot fears. URL: <https://www.cnbc.com/2021/03/15/covid-ireland-netherlands-suspend-astrazeneca-vaccine-amid-blood-clot-fears.html>
- 2021-03-25: Biden Announces New Vaccine Goal: 200 Million Shots in 100 Days. URL: <https://www.usnews.com/news/health-news/articles/2021-03-25/biden-announces-new-vaccine-goal-200-million-shots-in-100-days>
- 2021-03-25: CDC Plans to Provide $332 Million to Support Community Health Workers for COVID-19 Prevention and Control. URL: <https://www.cdc.gov/media/releases/2021/p0325-community-healthworkers-support.html>
- 2021-03-25: F.D.A. Grants Emergency Authorization for Johnson & Johnson Vaccine. URL: <https://www.nytimes.com/live/2021/02/27/world/covid-19-coronavirus>
- 2021-03-29: More Than a Dozen States to Open Vaccines to All Adults. URL: <https://www.usnews.com/news/health-news/articles/2021-03-29/at-least-11-more-states-to-open-virus-vaccines-to-all-adults>
- 2021-04-07: UK variant is now the dominant coronavirus strain in the US, says CDC chief. URL: <https://edition.cnn.com/2021/04/07/us/uk-variant-dominant-coronavirus-strain/index.html>
- 2021-04-12: Two New Studies Show That the U.K. COVID-19 Virus Variant Is Not Linked to Severe Disease—But Questions Remain. URL: <https://time.com/5954287/latest-studies-show-uk-covid-19-virus-variant-is-not-linked-to-severe-disease-but-questions-remain/>
- 2021-04-15: Dr. Fauci: ‘We very well may need to get booster shots’ for Covid — here’s when. URL: <https://www.cnbc.com/2021/04/15/dr-fauci-when-we-will-need-booster-shots-for-covid-vaccine-timeline.html>
