# Supplementary material for: The Evolution and Disparities of Online Attitudes Toward COVID-19 Vaccines: Year-long Longitudinal and Cross-sectional Study
Source: J Med Internet Res. 2022 Jan 21;24(1):e32394. doi: 10.2196/32394 (PMC8786033; doi:10.2196/32394)
Supplement: Multimedia Appendix 2 [file jmir_v24i1e32394_app2.docx]

**Multimedia Appendix 2.** Supplemental information about the sentiments toward vaccines at 5 major vaccine-related events.

Table S1 in this appendix shows the sentiment polarities of population groups at the five major vaccine-related events. From this table, we can see that after the inoculation started worldwide (at *t*_4_ and *t*_5_), the following population groups always carried lower sentiments under the six demographic characteristics: individuals, males, people with age ≤18, age ≥40 years old, OC3 (occupations of the 3rd category), account age <5, and follower number <500.

**Table S1.** Sentiment polarities towards COVID-19 vaccines at the five major vaccine-related events. The lowest sentiment in each characteristic group is marked bold.

| Characteristic | Mean sentiment polarity towards COVID-19 vaccines | | | | |
| --- | --- | --- | --- | --- | --- |
|  | *t*_1_: 2020-07-14 | *t*_2_: 2020-08-12 | *t*_3_: 2020-11-09 | *t*_4_: 2020-12-15 | *t*_5_: 2021-04-10 |
| **User type** | | | | | |
| Individual | **0.0426** | **0.0112** | **0.0402** | **0.0192** | **0.0371** |
| Organization | 0.0946 | 0.0557 | 0.0602 | 0.0576 | 0.0371 |
| **Gender** | | | | | |
| Male | 0.0527 | 0.0129 | **0.0393** | **0.0167** | **0.0285** |
| Female | **0.0299** | **0.0095** | 0.0416 | 0.0227 | 0.0519 |
| **Age** | | | | | |
| ≤18 | **0.0155** | **0.0019** | 0.0941 | **0.0047** | 0.0375 |
| 19-29 | 0.0612 | 0.0214 | 0.0682 | 0.0217 | 0.0408 |
| 30-39 | 0.0337 | 0.0067 | 0.0490 | 0.0191 | 0.0450 |
| ≥40 | 0.0399 | 0.0085 | **0.0064** | 0.0088 | **0.0270** |
| **Occupation** | | | | | |
| OC1 | 0.0679 | 0.0205 | 0.0473 | 0.0283 | 0.0477 |
| OC2 | 0.0236 | 0.0066 | 0.0532 | 0.0130 | 0.0323 |
| OC3 | **0.0227** | **-0.0007** | **0.0193** | **-0.0037** | **0.0254** |
| **Account age** | | | | | |
| <5 | 0.0461 | **0.0014** | 0.0386 | **0.0144** | **0.0277** |
| 5-10 | 0.0517 | 0.0169 | **0.0200** | 0.0237 | 0.0413 |
| ≥10 | **0.0361** | 0.0328 | 0.0708 | 0.0344 | 0.0504 |
| **Follower number** | | | | | |
| <500 | 0.0510 | 0.0151 | 0.0364 | **0.0175** | **0.0298** |
| 500-5000 | 0.0423 | **0.0045** | **0.0236** | 0.0240 | 0.0457 |
| ≥5000 | **0.0393** | 0.0195 | 0.1019 | 0.0413 | 0.0578 |
